# Commentator Discussion: Heart transplant survival and the use of donors with intracranial bleeding: United Network for Organ Sharing registry propensity-matched analysis

**DOI:** 10.1016/j.xjon.2024.10.017

**Published:** 2024-10-26

See Article page 306.

Presenter: J. Sam Meyer

**Dr Pedro Catarino***(Los Angeles, Calif)*. Thank you to the association for the invitation to discuss this paper, and thank you to Mr Meyer, Dr Barak, and colleagues for sending me the manuscript in advance. Mr Meyer and colleagues are to be congratulated on this large analysis of United Network for Organ Sharing (UNOS) registry recipients in the predonation after cardiac death era. I have 3 questions, one donor-related, one recipient-related, and one general. As you so convincingly presented, there have been several studies that have addressed this question in the past, and so we know that intracranial bleed heart donors are typically older, more often female, smokers, diabetic, and hypertensive, and your study uses propensity matching to adjust for a number of important factors, but among these, really only donor age is included as far as the donor factors. Are the others not important? And why does just adjusting for age remove the adverse effect of donor intracranial bleed on long-term outcome?

**Figure:**
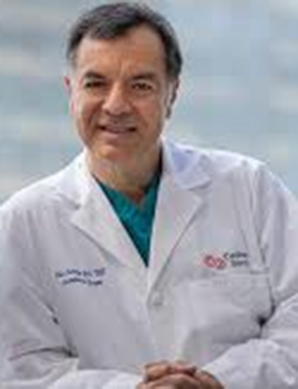

**Mr J. Sam Meyer***(Tel Aviv, Israel)*. Thank you very much for taking the time to review the paper, and for your very thoughtful questions. If you could just go back one slide, please, sir, because I have some notes on there. Okay, so we're going to wing it. First, I will say you're right. As we showed, age really was the most important and maybe only statistically significant variable, but I think that if you look back at our methodology, the other variables independently maybe didn't show that they were statistically significant. Only younger age showed that it really moved the needle, but they likely had some kind of multifactorial effect. I can't say that the donor who had diabetes maybe was better or worse, but likely, the donor who had diabetes may have also had hypertension and also had been on insulin for many years, and so on and so forth. So, as we said, I think we have to do a deeper dive into the independent variables, and there are more that weren't included, obviously.

**Figure:**
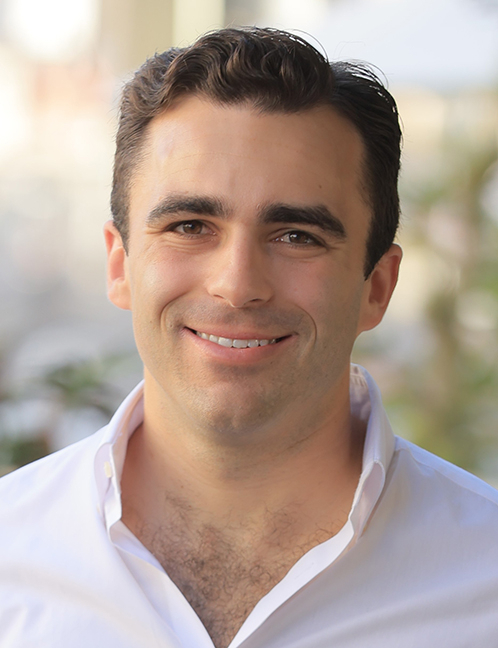

**Dr Catarino**. Okay. My second question, then, is about the recipients and whether we really believe that intracranial bleed donors are worse. So, the donor recipient matching is an art at the core of any successful transplant program. The transplant commission gets an offer for a particular recipient, then decides whether to use it or hold off for a better offer. So, I think the waiting time of the recipients can give some insight into what the transplant commissions think of the donors. And so, what I would like to know is do you know how long your recipients waited in each of the matched groups?

**Mr Meyer**. So, in the nonmatched groups, the waitlist was the—the waitlist time was same. So, therefore, we didn't continue it into the matched groups. But I'll say from just the clinical experience that is in Israel, because there isn't that privilege of waiting for the next transplant we got, although it kind of goes against my thesis, single-center, very small power, the outcomes are the same and we do use them. But yeah.

**Dr Catarino**. Okay, thank you. And so finally, bringing it together, cardiac dysfunction following brain injury is a topic of particular interest in this sort of postdonation after cardiac death era. Does your work mean we can ignore this, or do we need to account for other factors such as the timing of procurement in relation to the brain injury?

**Mr Meyer**. So, thank you. I first want to humbly say that we can't ignore anything and second, I want to say that I think more so than showing the importance of intracranial bleeding and traumatic brain injury (TBI) here, was showing the importance of propensity matching and showing the importance of maybe moving away from traditional statistical analyses and moving more towards propensity matching. So, to answer your question, I don't think we can ignore anything. My first instinct would say, “Let's take all the things that we want to ignore and match them first so that we're comparing apples and apples.”

**Dr Catarino**. Thank you.

**Mr Meyer**. Thank you.

**Unidentified Speaker 1**. Congratulations again. I think it is an excellent paper, and I certainly think that we should use TBI donors. If I can offer a gentle critique, the statistics are right. Your description is not correct, that propensity matching is that much better than multivariable regression, for example. In reality, it gives you the same result. It's just propensity matching is easier to describe and makes for prettier Kaplan-Meier curves. But if you run an appropriate multivariable regression model, it's the same thing, I'd say.

**Mr Meyer**. Thank you.

**Unidentified Speaker 2**. TBI and intracranial hemorrhage from a stroke that is a hemorrhagic conversion are very different donors. So, you put them all in the same group?

**Mr Meyer**. No. The point of the study was to separate them, actually. In a previous study, we looked at TBI. And here, instead of kind of using the same implications for a cohort of intracranial bleeding, we said we should do the same analyses on that cohort independently. That if—

**Unidentified Speaker 2**. Okay.

**Dr Erin Schumer***(St Louis, Mo)*. Erin Schumer from Barnes Jewish. So, your study went to 2018, which was before allocation change. Did you—

**Mr Meyer**. That was in—yeah, sorry, 2018.

**Dr Schumer**. So, did you look at the study after the allocation change? Because it would be interesting to see if that made any difference because our recipients are quite different from between the 2 system changes.

**Mr Meyer**. For sure. So, we didn't, yet. At our laboratory, we're doing a lot of UNOS database stuff, and the most important thing about doing UNOS database work is constantly re-updating it because the data get exponentially better every year, and the technologies are changing exponentially. So, every study that we're doing, every year as we get the data across the ocean, we rerun it.

**Unidentified Speaker 2**. Thank you.

**Mr Meyer**. Thank you.

[applause]

## Conflict of Interest Statement

The authors reported no conflicts of interest.

The *Journal* policy requires editors and reviewers to disclose conflicts of interest and to decline handling or reviewing manuscripts for which they may have a conflict of interest. The editors and reviewers of this article have no conflicts of interest.

